# Successful Autologous Blood Patch Pleurodesis in a Case of a Refractory Primary Spontaneous Pneumothorax

**DOI:** 10.7759/cureus.85344

**Published:** 2025-06-04

**Authors:** Shahnawaz Hashmi, Saquib Siddiqui, Lwin Paing, Harsh V Chawla

**Affiliations:** 1 Internal Medicine, South Tees Hospitals NHS Foundation Trust, Middlesbrough, GBR; 2 Respiratory Medicine, James Cook University Hospital, Middlesbrough, GBR; 3 Cardiology and Medicine, University Hospitals Bristol and Weston NHS Foundation Trust, Bristol, GBR

**Keywords:** autologous blood patching, autologus blood pleurodesis, blood patch, pneumothorax, refractory pneumothorax

## Abstract

Autologous blood patch pleurodesis (ABPP) is a novel therapeutic option for persistent/refractory pneumothorax (PTX) in patients who fail to respond to the initial thoracostomy procedure. Previous evidence from the literature suggests its successful implementation in secondary spontaneous PTX. We present our experience with autologous blood pleurodesis being used successfully in a primary spontaneous PTX. It also highlights the use of autologous blood pleurodesis in failed talc pleurodesis. We present a 33-year-old gentleman with a complex cardiac history who developed a large left-sided primary spontaneous PTX. He was initially treated with a chest drain. He then underwent chemical pleurodesis with talc, as the PTX was unresolved despite having a chest drain inserted for over five days. It was discussed with the cardiothoracic team, but due to his complex cardiac history and poor cardiac functional status (ejection fraction of 25%), the surgical team deemed him not safe for any surgical intervention. Talc pleurodesis failed in the resolution of the PTX. Therefore, autologous blood pleurodesis was performed, which showed complete resolution within 24 hours. This case highlights the potential effectiveness of ABPP for persistent PTX in primary spontaneous PTX.

## Introduction

Persistent air leaks (PALs) in pneumothorax (PTX), after chest drain insertion, remain a significant clinical challenge, often resulting in prolonged hospital stays and an increased risk of infection. Management becomes challenging for patients who are unsuitable for surgical intervention. Medical therapeutic options for such patients are chemical pleurodesis (talc pleurodesis), autologous blood pleurodesis, or endobronchial valves [1,2]. Autologous blood patch pleurodesis (ABPP) is a simple, safe, cost-effective, and well-tolerated procedure with a favourable outcome, such as shorter length of hospital stay, lower incidence of additional invasive procedures, and reoperations. We present one such case of refractory primary spontaneous PTX successfully treated with ABPP.

## Case presentation

**Figure 1:**
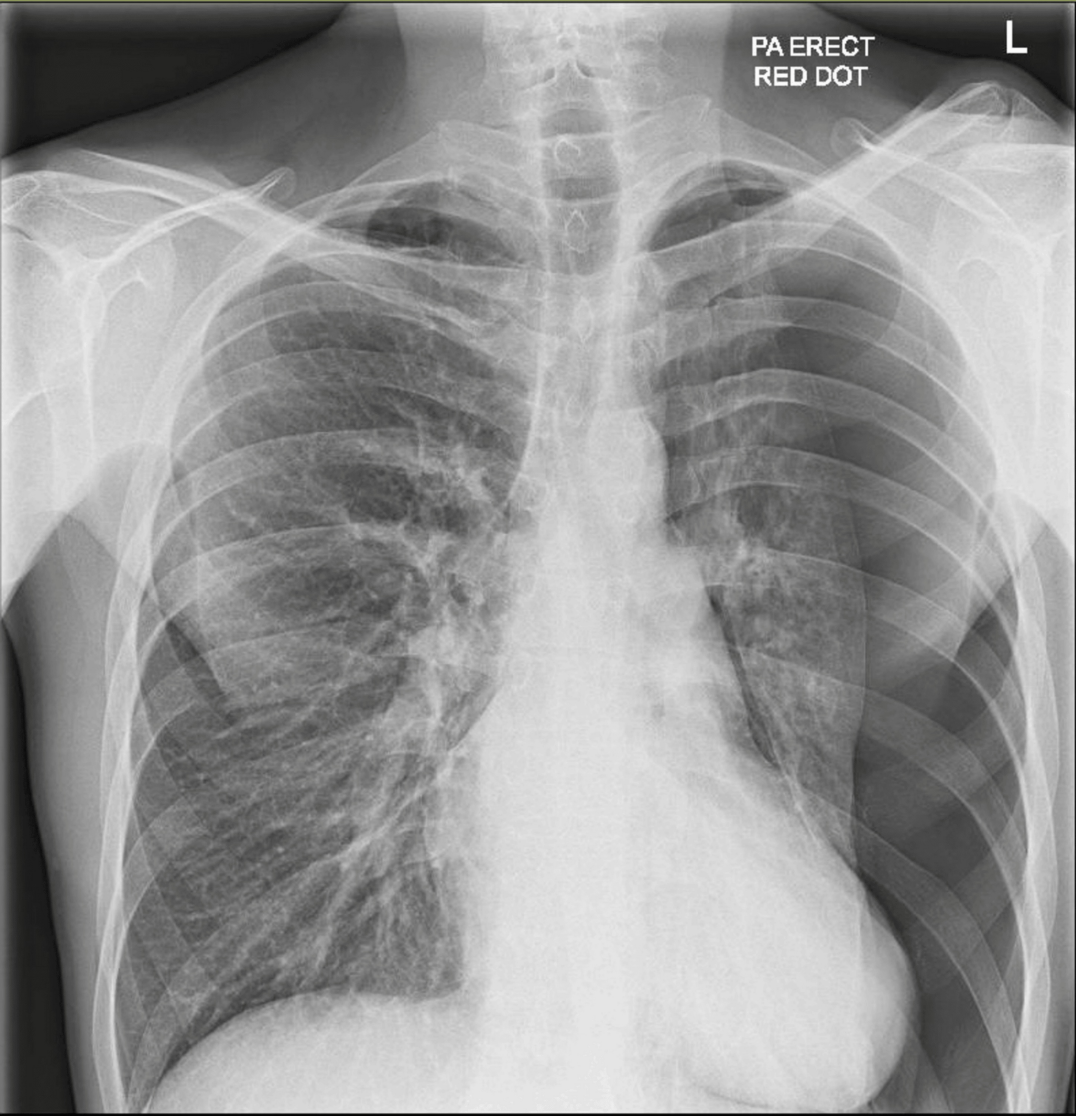
Chest X-ray on arrival showed a large left-sided pneumothorax

A 33-year-old gentleman, with a past medical history of myocardial infarction, chronic kidney disease, end-stage biventricular failure, atrial septal defect, ventricular thrombus, and a history of intravenous drug use (IVDU), presented to the emergency department (ED) with sudden onset chest pain and shortness of breath. He had a chest X-ray (CXR) in the ED, which revealed a large left-sided PTX (Figure 1). Subsequently, a 12-French chest drain was inserted, and the patient was moved to the acute medical ward for monitoring. He had no prior history of such. 

**Figure 2:**
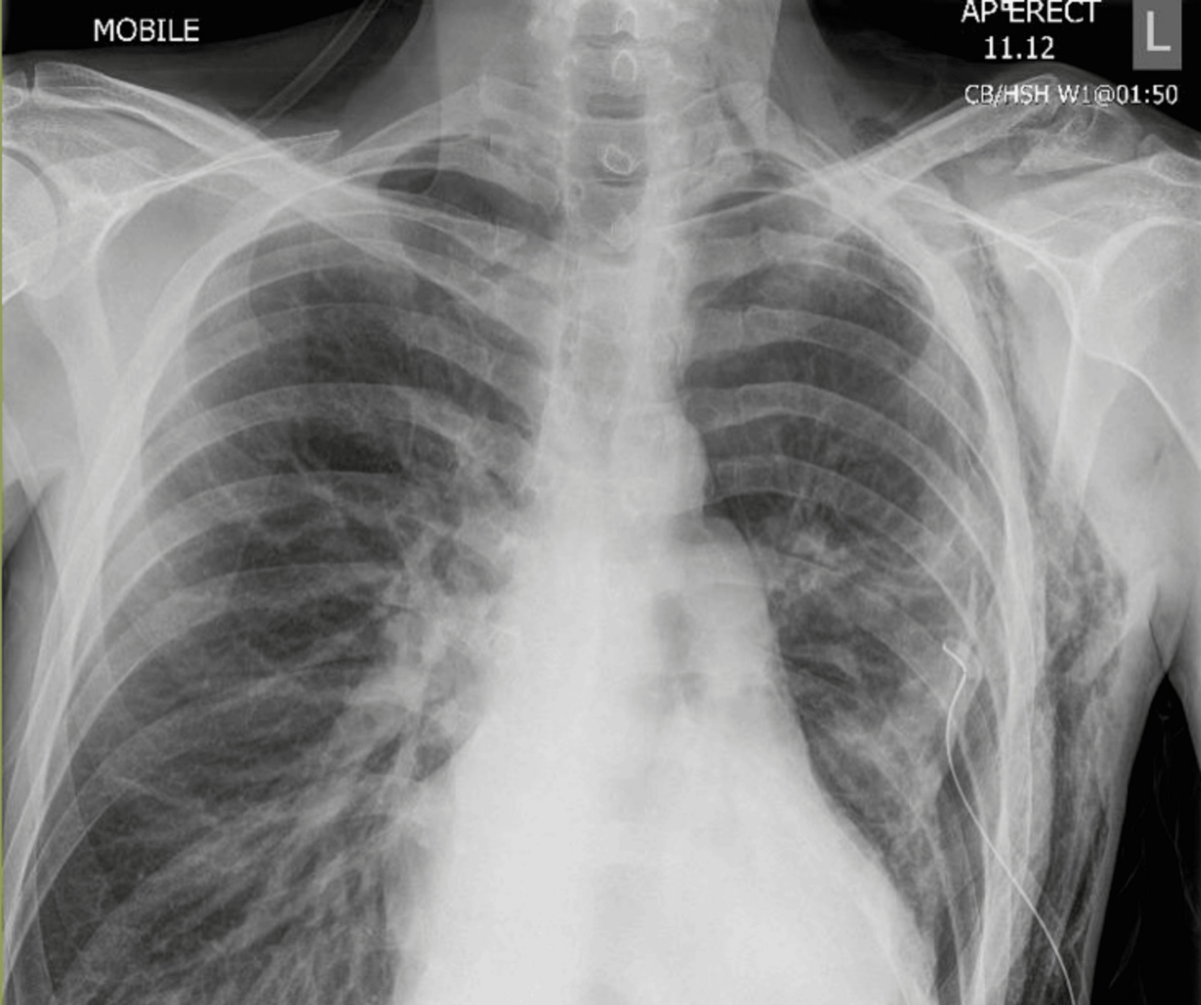
Chest X-ray showed the refractory left-sided pneumothorax despite chest drain insertion day 5

Repeat chest X-ray after five days showed a persistent PTX despite having a chest drain (Figure 2). Therefore, in view of the refractory PTX, we discussed with the cardiothoracic team for consideration of surgical intervention (VATS). Cardiothoracic deemed him unfit for surgery, looking at his poor cardiac functional status. The chest drain was put into suction -1 to -2 kPa for the following two days. Once there was adequate re-expansion of the lung, talc pleurodesis was performed. However, it did not resolve the PTX, and the patient remained symptomatic for the following five days post-talc pleurodesis.

**Figure 3:**
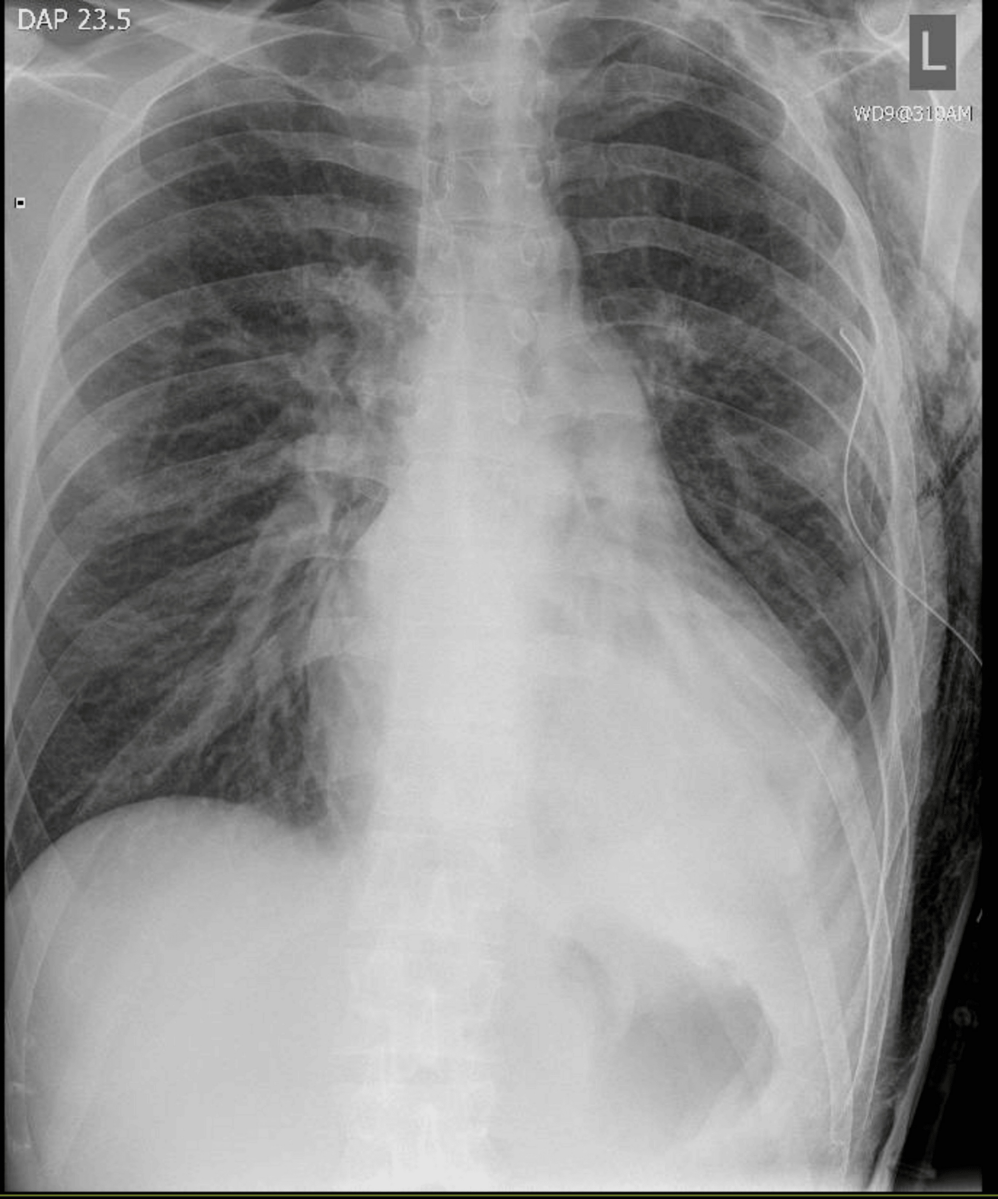
Chest X-ray showed resolution of the left-sided pneumothorax following blood pleurodesis

Therefore, autologous blood pleurodesis was performed, which showed resolution of the PTX within 24 hours (Figure 3).

Although the patient recovered from the PTX point of view, with symptomatic improvement alongside breathing comfortably on room air, he later died from multi-organ failure as he developed severe acute kidney injury (AKI) during admission, resulting in various electrolyte derangements, which eventually resulted in cardiac arrhythmia and death.

## Discussion

ABPP is a novel concept in treating persistent/refractory PTX. As per recent BTS guidelines, therapeutic options for refractory PTX are talc pleurodesis, autologous blood pleurodesis, endobronchial valve, and video-assisted thoracoscopy (VATS) [1,2].

ABPP is an effective interventional option for PTX, particularly for patients who are not suitable for surgical intervention due to poor lung reserve or other significant comorbid conditions. Previous literature supports its successful use in secondary spontaneous PTX [3], but there is no evidence of its use in primary spontaneous PTX. First introduced nearly 30 years ago, various theories have been proposed to explain its mechanism of action. One suggested mechanism is that blood induces pleurodesis through an intrapleural inflammatory reaction, while another theory posits that blood seals a leaky airway by forming a clot [3,4,5].

In the UK, the British Thoracic Society (BTS) is the primary body responsible for establishing guidelines on respiratory conditions. Most recent BTS guidelines have included ABPP as a therapeutic option for refractory PTX not amenable to surgical intervention [1,2]. 

Siddiqui et al., Shakir et al., and Metaxas et al. previously published their finding based on case series, and their study found ABPP as a safe and effective therapeutic option for patients who are not candidates for surgery [3,5,6]. All existing evidence proves its effectiveness in secondary spontaneous PTX. In our case, we have found it to be successful and effective in primary spontaneous PTX, too. To our knowledge, this is the first instance being reported as such. 

A key benefit of ABPP over chemical pleurodesis is that talc pleurodesis requires close apposition of the parietal and visceral pleura, which may not occur if the air leak remains significant. Also, it is a lot less painful procedure compared to talc pleurodesis/ One of the major issues or side effects of talc pleurodesis is that patients experience significant pain despite the application of local and systemic analgesics. 

ABPP has a lower risk of side effects. Previously documented side effects are infections and the development of tension PTX from a blocked chest drain due to a blood clot [3,5,7]. This can be prevented by extending the intercostal chest drain tube and creating a loop resting it above the level of the patient’s body (as described in the BTS guidelines). Moreover, Siddiqui et al. in their study mentioned using normal saline or sterile water flushes in larger amounts of 150-200 ml after instilling blood to ensure that drain patency remains intact and the chest drain tube is not blocked with clots [8].

## Conclusions

ABPP is a viable and safe therapeutic option for PTX with PALs. It has been previously found effective in secondary spontaneous PTX. Our experience suggests that it is an excellent and safe therapeutic means to treat refractory primary spontaneous PTX, too.
